# Hemodynamic Collapse Due to Unrecognized Hemothorax Following Central Venous Catheter Insertion in a Resource-Limited Setting: A Case Report

**DOI:** 10.7759/cureus.81929

**Published:** 2025-04-08

**Authors:** Gegal Pruthi, Jyoti Kanwat, Ruhi Sharma, Gopal Jalwal

**Affiliations:** 1 Anaesthesiology, All India Institute of Medical Sciences, Bathinda, IND

**Keywords:** central venous catheterization, cvc, hemothorax, internal jugular vein, ultrasound

## Abstract

Central venous catheter (CVC) placement is a routine but not risk-free procedure, with potential complications including hemothorax. We present a case of a 71-year-old male with coronary artery disease (CAD) who developed intraoperative hemothorax following internal jugular vein CVC placement. The patient experienced profound hypotension during surgery, prompting intervention. Subsequent exploration revealed a small abrasion on the right lung surface caused by an inadvertent initial CVC puncture, leading to hemothorax. The patient was successfully resuscitated, and prompt identification and management of hemothorax were critical. This case emphasizes the importance of vigilance, communication, and early consideration of complications like hemothorax post-CVC placement when unexplained hemodynamic instability occurs. We believe that the use of ultrasonic guidance or fluoroscopic guidance during CVC placement could reduce unusual major mechanical complication rates even during emergencies.

## Introduction

Central venous catheter (CVC) placement is a routine procedure in high-risk cases, critically ill patients, and those with limited peripheral vascular access. While generally considered safe, it is not without risks, including arterial puncture, hematoma, pneumothorax, hemothorax, catheter infections, and thrombosis [1-4]. The use of ultrasound guidance has reduced the incidence of some complications [5,6]. However, unusual complications like intra-arterial cannulation, extravascular catheterization, or intravenous misplacement of the catheter tip can still occur [7]. This case report describes a challenging scenario in a 71-year-old male with coronary artery disease (CAD) who developed a life-threatening intraoperative complication, hemothorax, following internal jugular vein CVC placement.

## Case presentation

**Figure 1:**
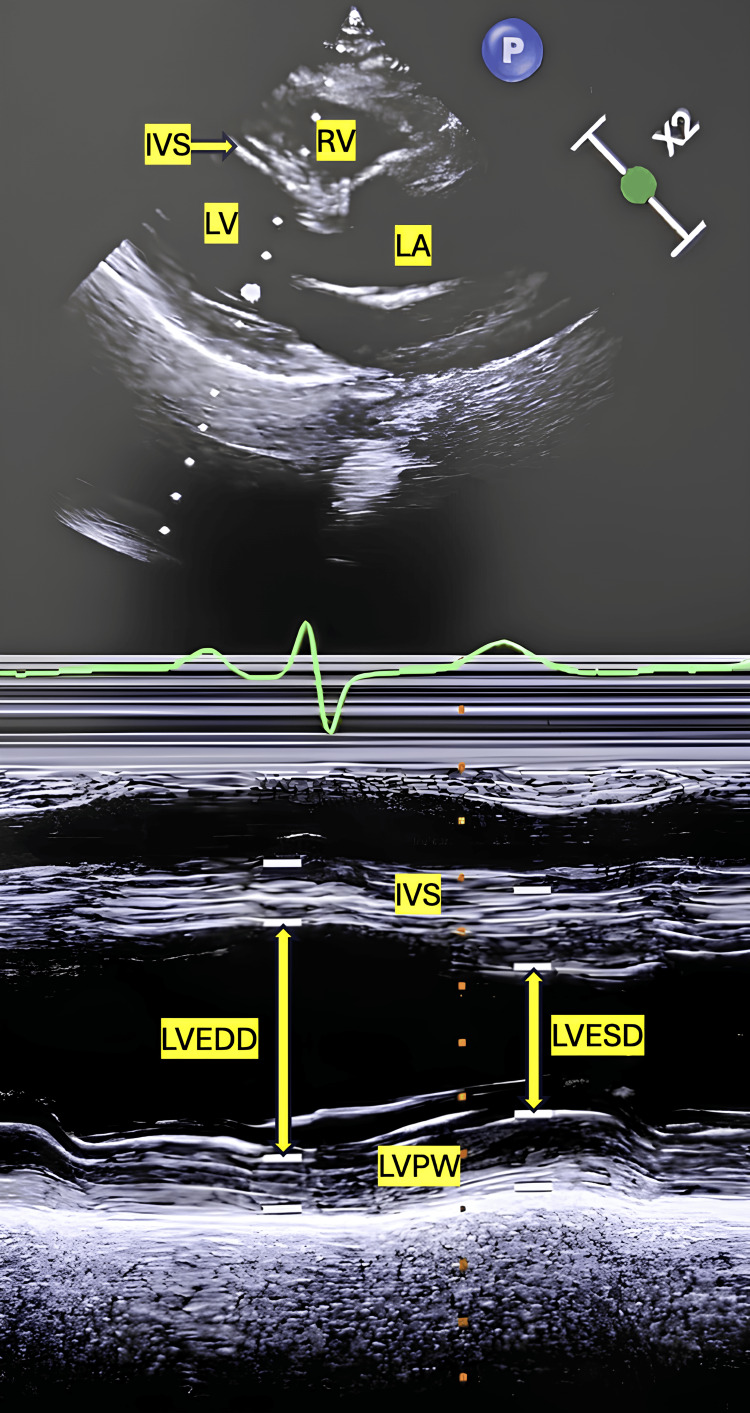
M mode measurement of left ventricular ejection fraction (LVEF): 50% LV- left ventricle, RA- right atrium, LA- left atrium, IVS- interventricular septum, LVPW- left ventricular posterior wall, LVEDD- left ventricular end-diastolic diameter, LVESD- left ventricular end-systolic diameter

A 71-year-old male (70 kg, 156 cm) presented with complaints of chest pain and exertional dyspnoea. He was diagnosed with CAD and had a preoperative echocardiogram showing basal posterior, basal interventricular septum, and basal inferior wall akinetic segments. Additionally, the echocardiography revealed mild tricuspid regurgitation and mild left ventricular systolic dysfunction with an ejection fraction (EF) of around 50% (Figure 1). The patient had significant CAD, with a diffuse calcified lesion in the proximal left anterior descending artery and near-total proximal occlusion of the right coronary artery. Preoperative management included nitroglycerin and antiplatelet therapy. The patient had no other comorbidities, addictions, or significant family history. Laboratory investigations were within normal limits, except for a platelet count of 80,000/cumm. After taking informed consent, the patient was prepared for off-pump coronary artery bypass grafting and underwent standard monitoring. After local anesthesia, a peripheral 18G cannula was secured, and a 20G arterial cannula was inserted into the left radial artery. Anesthesia was induced with fentanyl (250 mcg) and etomidate (10 mg) followed by vecuronium (6 mg) for muscle relaxation, and the airway was secured with an 8.0 mm endotracheal tube. Subsequently, the patient was positioned for CVC placement.

**Figure 2:**
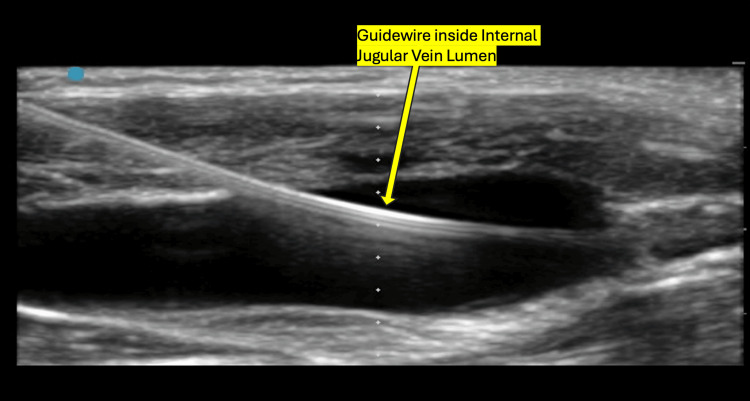
Long axis view of the internal jugular vein with the guidewire inside the lumen

Under strict aseptic precautions, the patient was positioned in a Trendelenburg position with the head slightly turned to the left to optimize the visualization of the right internal jugular vein (IJV). Using a high-frequency linear probe, ultrasound guidance was employed to identify vascular structures and confirm the patency of the right IJV. The initial needle insertion was performed using the out-of-plane technique, and free venous aspirate was obtained, confirming proper vessel entry. A guidewire was then carefully threaded through the needle, and its correct intravascular placement was confirmed with ultrasound (Figure 2). However, upon advancing the 7 French triple-lumen catheter over the guidewire, there was no free aspirate from any of the catheter ports, raising concerns about possible malposition or kinking. To ensure correct placement, the catheter was removed, and a second attempt was made at a slightly different angle under continuous ultrasound guidance. This time, free backflow was confirmed from all three lumens, and the guidewire was visualized within the vessel. After securing the catheter in place, it was flushed with saline, and appropriate dressing was applied. Airway pressures and hemodynamic parameters remained stable. The patient was then handed to the surgical team for the planned procedure. 

Soon after the surgical positioning, the patient developed profound hypotension, requiring fluid boluses and high-dose inotropes (noradrenaline at 0.1 mcg/kg/min and adrenaline at 0.1 mcg/kg/min) to maintain blood pressure. An intra-aortic balloon pump was promptly placed by the surgical team to counter the hypotension and for adequate coronary perfusion. Intraoperative transthoracic echocardiography revealed good contractility and inferior vena cava collapsibility of more than 50%. There were no fresh ST changes.

**Figure 3:**
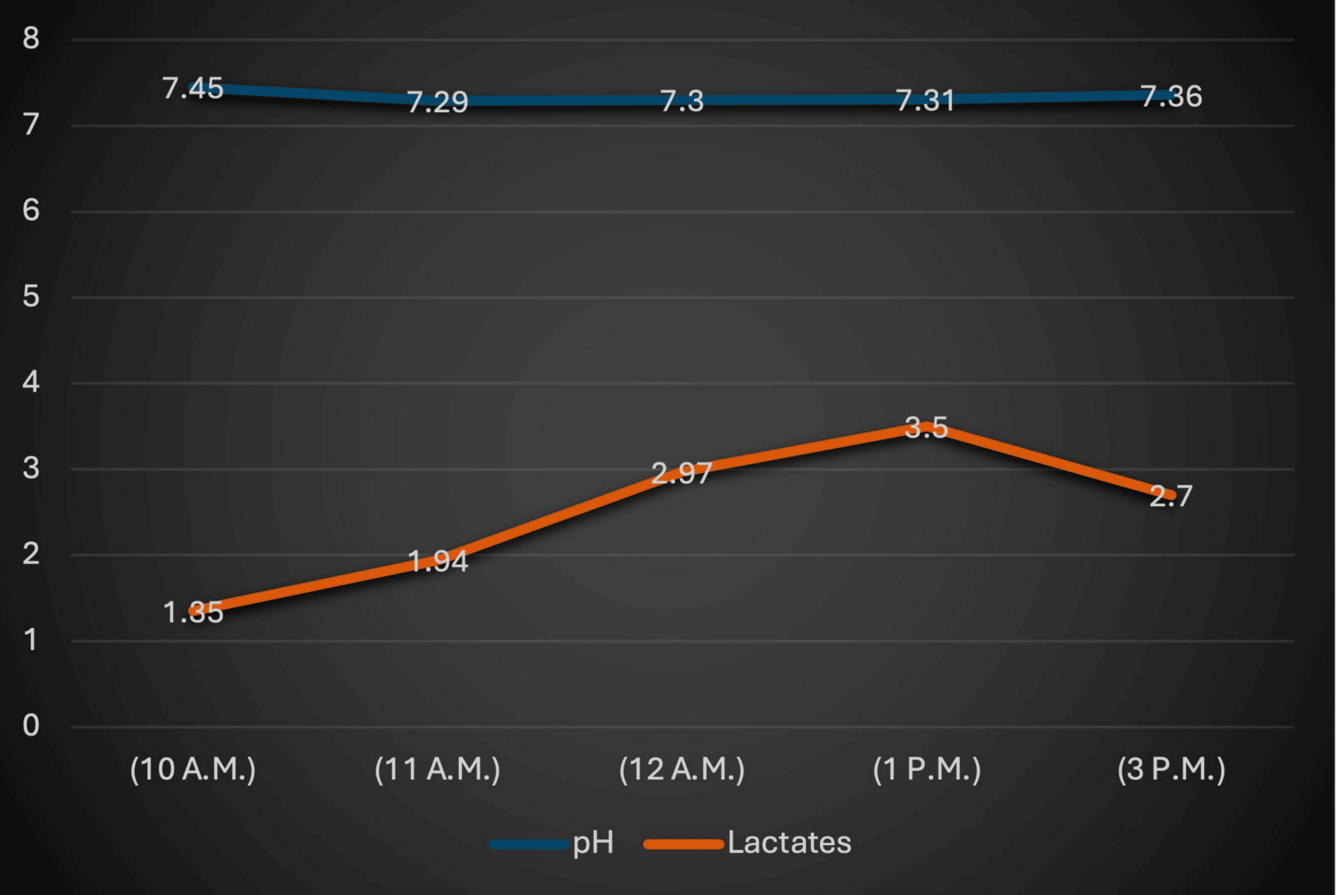
Intraoperative arterial blood gas parameters (pH and lactates)

The patient was resuscitated with fluid replacement and inotropes until hemodynamic stability was achieved. The surgical team then proceeded with grafting, including a left internal mammary artery to the left anterior descending artery and a right saphenous vein graft to the right coronary artery. Throughout the surgery, the patient required high-volume fluid replacement, inotropic support (infusion noradrenaline at 0.1 mcg/kg/min and infusion adrenaline at 0.1 mcg/kg/min), and blood transfusion to maintain a mean arterial pressure (MAP) of more than 65 mmHg. Serial arterial blood gas (ABG) analysis revealed increasing lactates and metabolic acidosis (Figure 3).

During final hemostasis after revascularization, bleeding was observed from the apex of the pleura, and further examination revealed collected blood clots behind the right lung.

Upon exploration, a small abrasion was observed on the surface of the right lung, identified as the source of the bleeding. The abrasion was cauterized, hemostasis was achieved, and the chest was closed. Immediately after controlling the source of bleeding, which was because of an inadvertent CVC puncture to the pleura, the patient’s hemodynamic parameters started to improve. Inotropes were carefully titrated to maintain a MAP above 65 mmHg, inotropic support was minimal toward the end of the surgery, and the patient was shifted to the intensive care unit in stable condition. Serial ABG measurements were performed with appropriate corrections, and a urine output of 600 ml was noted over four hours of surgery.

The patient experienced a total blood loss of approximately 2 liters during the procedure, which was replaced with 4 liters of crystalloids, 2 units of packed red blood cells, 4 units of fresh frozen plasma, and 4 units of random donor platelets.

## Discussion

Although ultrasound guidance has significantly improved the accuracy and safety of CVC line placement, complications can still arise despite adhering to best practices. Hemothorax, as observed in our case, is a recognized complication of IJV catheter placement, often caused by misplacement of the guidewire or other technical errors during the procedure [8-11]. Despite the low incidence of this complication, its life-threatening nature necessitates vigilant attention during insertion [3].

The misplacement of the guidewire can lead to unintentional entry into incorrect vessels such as the right subclavian vein or the azygos vein. Meticulous attention to guidewire positioning is imperative to prevent dilator-induced vein injury.

Hemothorax may not manifest immediately post-insertion, complicating its diagnosis [12,13]. In this case, suspicion arose after the initial inability to aspirate blood from the catheter ports. Subsequent reinsertion confirmed backflow from all three ports, providing an initial stability window. However, the patient's sudden hypotension prompted immediate intervention, including fluid resuscitation, inotropic support, and an intra-aortic balloon pump to stabilize the underlying coronary artery disease.

In our resource-limited setting, we performed transthoracic echocardiography (TTE) rather than transesophageal echocardiography (TEE), as a TEE probe was not available. According to guidelines from the American Society of Anesthesiologists and the Society of Cardiovascular Anesthesiologists (2010), TEE should be used in all open-heart and thoracic aortic surgical procedures and is recommended for consideration in coronary artery bypass graft surgeries [14]. Given the patient’s CAD and ongoing cardiac surgery, initial differentials for hemodynamic instability included myocardial ischemia or hypovolemia. Intraoperative transthoracic echocardiography ruled out cardiac dysfunction, and despite fluid resuscitation, persistent hypotension suggested an alternate cause. Stable airway pressures made pneumothorax unlikely, though an early lung ultrasound could have facilitated diagnosis. Although TEE offers superior visualization, particularly for detecting right-sided pleural effusion in cases of massive hemothorax, we relied on TTE (because of the non-availability of TEE) for intraoperative hemodynamic assessment. The delayed onset of hypotension and visual bleeding source ultimately raised suspicion of CVC-related complications, leading to the identification of hemothorax.

In hindsight, effective communication between the anesthesia and surgical teams could have played a pivotal role in identifying catheter malposition during the initial insertion. Such collaboration might have raised suspicion of potential complications, prompting earlier investigation and intervention. Additionally, considering hemothorax as a cause for unexplained hemodynamic instability is crucial in the absence of routine intraoperative chest X-rays post-CVC insertion. Catheter malposition can also be detected using fluoroscopy by well-trained radiologists, which could aid in the early identification and prevention of complications.

Unlike the presented case, a report by Hyun Kang highlighted persistent intraoperative hypotension with hemothorax identified postoperatively [8]. This emphasizes the importance of proactive monitoring, especially in high-risk procedures.

## Conclusions

This case underscores the importance of vigilance and prompt intervention in managing rare complications like hemothorax during CVC. Despite ultrasound guidance, procedural risks remain, necessitating careful techniques and real-time monitoring. Sudden intraoperative hypotension should prompt suspicion of vascular injury, particularly in high-risk patients. Effective communication between anesthesiologists and surgeons is crucial for early recognition and management. Heightened awareness and preparedness can improve patient safety and surgical outcomes. The authors believe that the use of ultrasonic guidance or fluoroscopic guidance during CVC placement could reduce unusual, major mechanical complications even during emergencies.
